# The Thyroid Receptor Modulator KB3495 Reduces Atherosclerosis Independently of Total Cholesterol in the Circulation in ApoE Deficient Mice

**DOI:** 10.1371/journal.pone.0078534

**Published:** 2013-12-04

**Authors:** Lisa-Mari Mörk, Stefan Rehnmark, Padideh Davoodpour, Giuseppe Danilo Norata, Lilian Larsson, Michael-Robin Witt, Johan Malm, Paolo Parini

**Affiliations:** 1 Clinical Chemistry, Department of Laboratory Medicine, Karolinska Institute, Stockholm, Sweden; 2 KaroBio AB, Stockholm, Sweden; 3 Department of Pharmacological Sciences, Università degli Studi di Milano, Milan, Italy; 4 Molecular Nutrition Unit, Department of Biosciences, Karolinska Institutet, Stockholm, Sweden; University of Padova, Italy

## Abstract

**Background:**

Thyroid hormones (TH) regulate cholesterol metabolism but their use as lipid-lowering drugs is restricted due to negative cardiac effects. TH mimetic compounds modulating TH receptor β (THRβ) have been designed as potential drugs, reducing serum cholesterol levels while avoiding apparent deleterious cardiac effects.

**Objective:**

Using ApoE deficient mice, we examined whether KB3495, a TH mimetic compound, reduces atherosclerosis and if there is a synergistic effect with atorvastatin. The effect of KB3495 was investigated after 10 and 25 weeks.

**Results:**

KB3495 treatment reduced atherosclerotic plaque formation in aorta and decreased the cholesteryl ester (CE) content by 57%. Treatment with KB3495 was also associated with a reduction of macrophage content in the atherosclerotic plaques and reduced serum levels of IL-1β, TNFalpha, IL-6, Interferon γ, MCP-1 and M-CSF. Serum lipoprotein analysis showed no change in total cholesterol levels in ApoB-containing lipoproteins. KB3495 alone increased fecal BA excretion by 90%. The excretion of neutral sterols increased in all groups, with the largest increase in the combination group (350%). After 25 weeks, the animals treated with KB3495 showed 50% lower CE levels in the skin and even further reductions were observed in the combination group where the CE levels were reduced by almost 95% as compared to controls.

**Conclusion:**

KB3495 treatment reduced atherosclerosis independently of total cholesterol levels in ApoB-containing lipoproteins likely by stimulation of sterol excretion from the body and by inhibition of the inflammatory response.

## Introduction

The first-choice treatment to decrease low density lipoprotein cholesterol (LDL-C) and reduce the risk for atherosclerosis are 3-hydroxy-3-methylglutaryl coenzyme A (HMGCoA) reductase inhibitors (statins) [1]. Statins lower hepatic cholesterol levels and thereby activate sterol regulatory element binding protein 2 (SREBP2) which in turn induce the expression of the LDL receptor (LDLR) resulting in increased LDL-C uptake from plasma. Newer drugs like ezetimibe, which acts by blocking intestinal cholesterol uptake, have recently been proposed as complements to statin therapy. Despite these new therapeutic approaches there still is a demand for improved treatment strategies. An approach currently widely debated is the enhancement of reverse cholesterol transport (RCT)[2], leading to an increased efflux of cholesterol from peripheral tissues and to a final excretion of cholesterol in the feces.

Thyroid hormone (TH) reduces circulating cholesterol levels [3,4] but the deleterious effects on the skeleton, the muscles and the heart prevents its use as lipid lowering drug [5,6]. TH binds to two distinct receptors, TRα and TRβ. TRα regulates heart rate whereas TRβ is highly expressed in the liver and plays a major role in regulating cholesterol metabolism [7,8]. More recently, TH mimetic compounds that specifically modulate TRβ either by selective hepatic uptake and/or by higher binding affinity to TRβ, have been designed as potential drugs, one compound has been tested in the clinic [9,10]. These substances have been shown to reduce serum cholesterol while avoiding apparent side-effects on the heart [11–13]. 

The present study investigates the combined effect of KB3495 [Thyroid Receptor Agonists, PCT: WO/05092317A1] a preferential TR β ligand, and atorvastatin on atherosclerosis. We choose to combine a preferential TR β ligand with a cholesterol synthesis inhibitor in order to detect possible additive and synergistic effects on cholesterol metabolism and atherosclerosis. This in light of the possibility to use TR β modulators as complement to statin therapy. ApoE deficient mice, an established mouse model for atherosclerosis, were treated with KB3495 and atorvastatin either alone or in combination for a period of 10 or 25 weeks. Following KB3495 treatment, atherosclerosis was markedly reduced and the effect was shown to be independent of total cholesterol levels in ApoB-containing lipoproteins and associated to a reduction of inflammatory response. KB3495 together with atorvastatin also reduced cholesterol synthesis, increased bile acid (BA) formation and induced excretion of fecal BA and neutral sterols.

## Materials and Methods

### Ethics Statement

Studies were approved by the institutional Animal Care and Use Committee at Stockholms södra djurförsöksetiska nämnd (Dnr S27-05).

### Studies with KB3495

TR-binding affinities were measured as described [14,15]. Briefly, KB3495 (Figure S1) was incubated with [^125^I]T_3_ and recombinant hTRα or –β until equilibrium and unbound ligand was separated from receptor-bound ligand. IC_50_ values represent the concentration of KB3495 inhibiting 50% of the binding of [^125^I]T_3_ (Figure S1).

### Animals

Eighty male ApoE-/- mice (Jackson Laboratories) were challenged with a western like diet containing 10% of calories as saturated fat and 0.2% cholesterol (w/w) (Harlan Laboratories). Food and water was given *Ad libitum*. The animals were divided into 4 groups, control group, KB3495 group receiving diet supplemented with 0.7 mg/kg (w/w) of KB3495, atorvastatin group receiving diet supplemented with 110 mg/kg (w/w) of atorvastatin and the combination group receiving diet supplemented with 0.7 mg/kg (w/w) of KB3495 and 110 mg/kg (w/w) of atorvastatin, corresponding to a total daily dose of approximately 3.5 µg KB3495 and 550 µg atorvastatin. 

Half of the animals in each group were sacrificed after 10 weeks and the rest following 25 weeks of treatment. Mice were fasted for 4 hours prior sacrifice. Blood was drawn by cardiac puncture under carbon dioxide anesthesia. Livers and intestines were frozen in liquid nitrogen. Skin and aorta were frozen in liquid nitrogen or alternatively stored in formalin. Feces were collected group-wise the last 48 h of the experiment in which the animals were sacrificed after 10 weeks treatment (10 w). 

### Histological investigation

Aortas from 6 animals in the control group (25 w) and 9 animals in the KB3495 group (25 w) were cryo-sectioned and stained for hematoxylin/eosin and oil red-O. Macrophages were detected using a Mac 1antibody (Pharmingen, BD). The samples were incubated with HRP labeled EnVision-rabbit (Dako) before counterstaining with hematoxylin. A plaque index was calculated by dividing the plaque area with the aorta circumference. 

Skin biopsies from 5 animals in the control and KB3495 group and 4 animals in the atorvastatin and combination group were collected after 25 weeks of treatment. Skin was paraffin embedded and thickness of dermis and epidermis was measured. Macrophages were detected using a Mac3 antibody and the percentage of dermis and epidermis covered with macrophages was determined. 

All areas were calculated by using Leica Q500, an image analysis system. The histological investigation was done by MicroMorph Histology Services, Lund, Sweden.

### Lipid Analysis

Size exclusion chromatography of serum lipoproteins from each individual mouse was performed according to Parini et al. [16]. Lipids from aorta, liver and skin from individual animals were extracted in chloroform/methanol [17]. Total and free cholesterol levels were determined by isotope dilution-mass spectrometry according to Björkhem I et al. [18] with some modifications, see supplements for details. 

Lathosterols were analyzed according to Lund et al. [19]. After lipid extraction remaining tissue was dissolved in 1N NaOH for protein determination. 

### Cytokine and Chemokime Analysis

Cytokines and chemokine in serum were measured using the Milliplex ® MAP Mouse Cytokine/Chemokine Magnetic Premixed Bead panel Immunoassay (Millipore) according to the manufacturer’s instructions. 

### Isolation of Hepatic Membranes and Analysis of Protein Expression

Hepatic membranes of individual animals were isolated and ABCA1, LDLR and SR-B1 protein levels were quantified with western blot, see supplementary data for a detailed protocol. Quantification of protein expression was done by titration of the loaded samples. By this approach, the slope of the curve describing the regression between the luminescence signal and the amount of protein loaded reflects the concentration of the protein of interest. 

### Isolation of Hepatic Microsomes and ACAT activity

Liver microsomes from individual animals were isolated and ACAT1 and ACAT2 activity was determined as described previously [20]. 

#### Gene Expression

RNA from individual animals was extracted with *Trizol reagent* (Invitrogen). cDNA synthesis was performed using MultiScribe Reverse Transcriptase (Applied Biosystems). Quantitative real-time PCR was performed with SYBR Green assay on an ABI Prism 7000 instrument (Applied Biosystems). As endogenous control the mean value of cyclophilin and GAPDH was used. *Primers span* over exon-exon junction, sequences are available on request.

### Bile Acid analysis

Feces were analyzed for neutral sterols and BAs according to Miettinen et al. and Grundy et al. [21,22] with some modifications. Most importantly, no column chromatography was used prior to gas chromatography.

### Statistics

Data are presented as means ± SEM. The significance of differences between groups was tested by ANOVA followed by post-hoc comparisons of group means according to the LSD method (Statistica software, Stat Soft Inc.). Contrasts between two groups were performed according to Student’s t test. Data were log-transformed when the assumptions of homoscedasticity and no correlation between means and variances were not met [23]. 

## Results

### Atherosclerosis development

**Figure 1:**
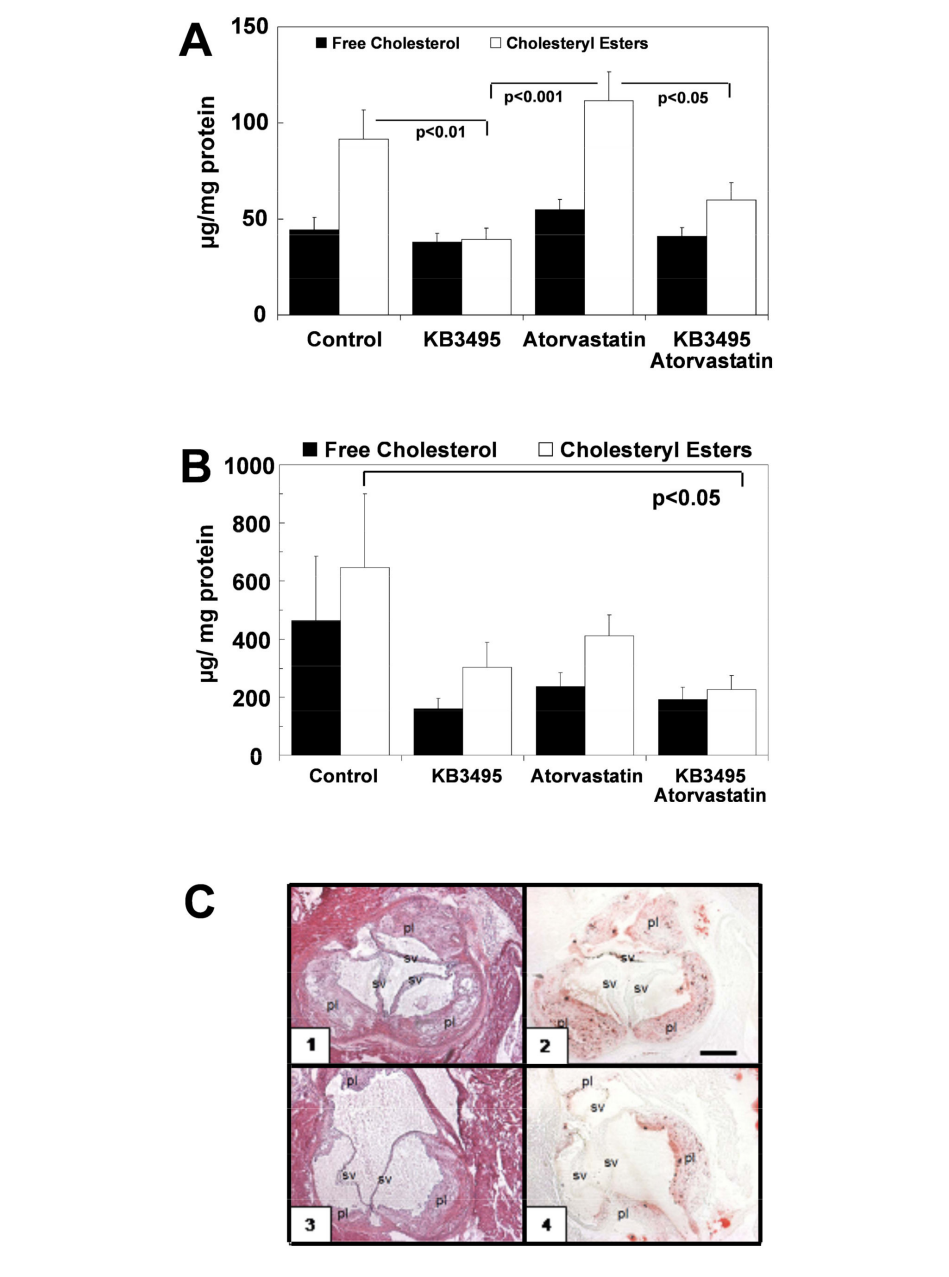
Cholesterol content in aorta. (A) Free cholesterol and cholesteryl ester content in the aorta from mice treated for 10 weeks, n=10 in control and KB group, n= 9 in Atorvastatin group and combination group. (B) Free cholesterol and cholesteryl ester content in the aorta from mice treated for 25 weeks, n=6 in controls, n=7 in KB group, n= 8 in Atorvastatin group and combination group. Values in (A) and (B) are corrected for protein levels and show mean ± SEM. (C) Hematoxylin/eosin (1,3) and oil red staining (2,4) of aorta from one control animal treated for 25 weeks (1, 2) and one animal treated with KB3495 for 25 weeks (3, 4). pl indicates plaque formation, sv indicates semilunar valves. Scalebar in 2 represents 300 μm and is valid for 1-4.

In order to evaluate the effects of long term treatment with the preferential TR β ligand KB3495 alone or in association with atorvastatin on the development of atherosclerosis ApoE deficient mice were treated for 10 or 25 weeks. After 10 weeks of treatment, 50% of the animals in each group were sacrificed and aortas collected. Atherosclerosis was measured by determination of lipid content in accordance to Rudel LL at al. [24]. Cholesteryl ester (CE) levels in the aortas were approximately 60% lower in the group receiving KB3495 (p< 0.01; Figure 1A) as compared to the control group. A tendency to reduced CE levels in the combination group (KB3495 + atorvastatin) was observed. When all the data relative animals treated with a specific treatment were analysed by 2-way ANOVA, it was clear that KB3495 significantly reduced the CE content in aorta. The effect was independent of the presence or absence of atorvastatin. Atorvastatin had no effect. Free cholesterol (FC) levels remained stable.

Next, we investigated the effects of the maintenance of the treatments for 25 weeks. Following 25 weeks CE levels in the aorta were reduced by 65% (p<0.05; Figure 1B) in the combination group. There was a trend towards reduced levels also in the KB3495 and the atorvastatin group; however this did not reach statistical significance using 1-way ANOVA. Nevertheless, KB3495 lowered the levels of CE in aorta (p<0.05) independently of the presence or absence of atorvastatin when the data were analyzed by 2-way ANOVA.

To investigate if atherosclerosis measured as CE content in the whole aorta corresponded to plaque size, aortic valves from KB3495-treated and control animals underwent histological examination and oil red-O staining (Figure 1C). The plaque index was reduced by 53% in KB3495 animals compared to controls (0.06 ± 0.01 *vs.* 0.13 ± 0.01; p< 0.05) and the percentage of oil red-O staining in the plaques was reduced by 35% (20.7% ± 3.69 *vs.* 13.4% ± 1.38; p<0.005). 

Staining also revealed that KB3495-treated animals had a lower percentage of macrophages in the plaques (p<0.05; Table 1). 

**Table 1:** Macrophage staining (%) in aorta and dermis after 25 weeks of treatment.

	**Aorta**	**Dermis**
**Ctrl**	48.4±2.2 (6)	22.6±3.1 (5)
**KB**	40.7±2.5* (9)	8±1** (5)
**Atorva**	Not measured	10.9±4.3** (4)
**Comb.**	Not measured	No detection (4)

Ctrl: controls; KB: treatment with KB3495; Atorva: treatment with atorvastatin; Comb.: treatment with KB3495 and atorvastatin in combination. (n): numbers of animals analyzed. Mean values ± SEM, contrast *vs.* Ctrl: *= *p*<0.05; ** = *p* < 0.01; *** = *p* < 0.001.

### Serum lipoproteins

In order to investigate if the observed reduction in the atherosclerotic lesions could be explained by lower levels of circulating cholesterol the serum lipoproteins were analysed. After 10 weeks of treatment, total serum cholesterol was not affected by KB3495 treatment alone but a minor decrease was detected in the combination group (p< 0.05; Table 2). After 25 weeks no difference between the groups could be detected. To investigate the lipoproteins in detail, the lipoprotein profiles were analyzed by SEC. In ApoE deficient mice lipoproteins consist mainly of chylomicron remnants (CR) of intestinal origin [25]. These particles separate within the size/density interval typical of VLDL and LDL particles making it impossible to isolate pure CR, VLDL and LDL fractions. Hence, we refer to these particles as ApoB-containing particles (intestinal and hepatic). See Table 2 and Figures S2 and S3 for lipoprotein profiles.

**Table 2:** Serum lipoproteins from animals treated 10 and 25 weeks.

		**Total**		**Free**		**Triglycerides**		**Phospholipids**	
		**cholesterol**		**Cholesterol**					
		**Total**	**AB**	**Total**	**AB**	**Total**	**AB**	**Total**	**AB**
**10 weeks**	**Ctrl**	13.78	13.35	4.46	4.46	0.88	0.74	13.50	13.49
	**KB**	14.82	14.27	5.93*	5.92*	1.22	1.14*	15.08	15.06
	**Atorva**	11.74	11.21	4.02	4.02	0.56	0.46	14.90	14.89
	**Comb.**	11.15*	10.70*	4.20	4.19	0.72	0.62	12.03	12.02
**25 weeks**	**Ctrl**	16.39	16.38	8.67	7.99	2.84	2.80	8.17	7.11
	**KB**	14.96	14.96	9.63	8.78	3.47	3.44	5.19***	4.40***
	**Atorva**	18.17	18.17	12.00***	11.18***	8.21***	8.15***	5.85**	5.33*
	**Comb.**	17.06	17.05	8.91	8.31	5.06*	5.01*	3.95***	3.47***

AB: apolipoprotein B-containing lipoproteins. Ctrl: controls; KB: treatment with KB3495; Atorva: treatment with atorvastatin; Comb.: treatment with KB3495 and atorvastatin in combination. Data is expressed in mmol/L, mean values are shown. In animals treated for 10 weeks n= 10 per group, in animals treated for 25 weeks n=7 in controls, n=10 in KB group, n= 9 in Atorvastatin group, n= 8 in combination group. Contrast *vs.* Ctrl * = *p* < 0.05; ** = *p* < 0.01; *** = *p* < 0.001.

After 10 weeks of treatment no major effects on TC levels in ApoB-containing particles were observed, only a small decrease in the combination group was detected. FC and TG increased in the ApoB-containing particles in mice receiving KB3495 alone, whereas the levels remained unchanged in the animals receiving atorvastatin alone or in combination with KB3495. Analysis of PL in the particles showed no difference between the groups. 

Following 25 weeks of treatment the TC levels in ApoB-containing lipoproteins were still not changed following KB3495 treatment and remained unchanged also following the other treatments. FC levels remained unchanged in the animals receiving KB3495 but were increased in mice receiving atorvastatin. Analysis of TG content in the lipoproteins revealed an increase in the atorvastatin and combination groups. Analysis of PL showed lower lipoprotein levels in all treatment-groups compared to controls. 

### Serum granulocytes and monocytes differentiation factors and inflammatory cytokines

Since treatment with KB3495 and atorvastatin was associated with reduced numbers of macrophages in the atherosclerotic lesions we decided to investigate whether this effect was also associated with an improved inflammatory profile. We focused the analysis in animals treated for 10 weeks as the longer treatment (25 weeks) was associated with skin wounds thus limiting the physiopathological relevance of cytokine and chemokine analysis at this time point. 

**Figure 2:**
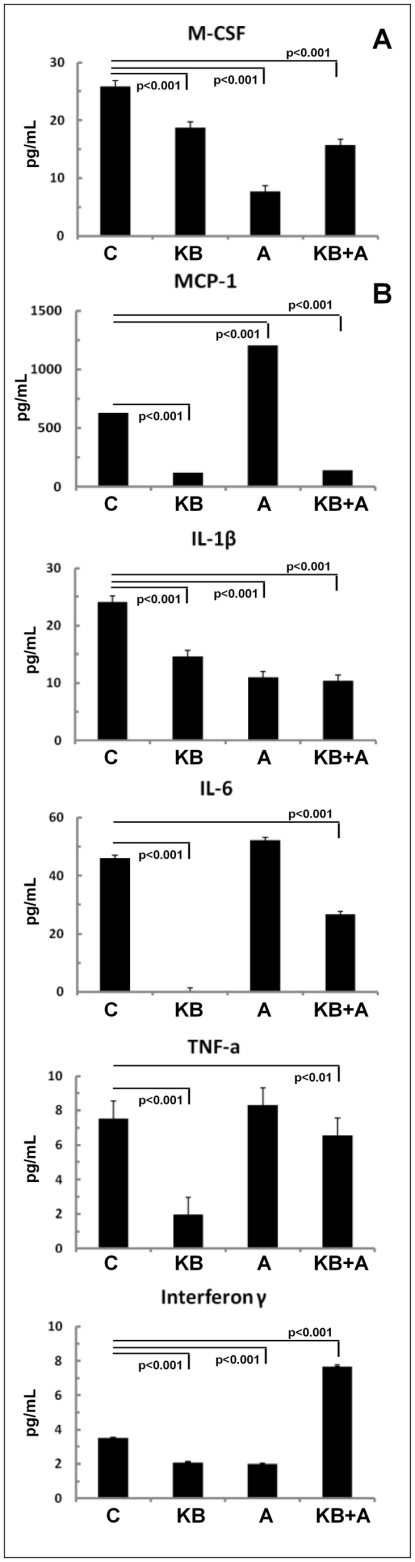
Serum profiling of granulocyte and monocyte differentiation factors and of chemokines and cytokines. Granulocytes and monocytes differentiation factors, cytokines and chemokines measured in sera, n=5 per group. Data show mean ± SEM for the control group. Panel 2A presents the data for granulocyte-macrophage colony-stimulating factor (GM-CSF). Panel 2B presents the data for monocyte chemoattractant protein 1 (MCP-1), interleukin (IL)-1beta, IL-6, tumor necrosis factor (TNF) alpha and interferon γ.

First, factors stimulating stem cells to differentiate into granulocytes and monocytes from the bone marrow, such as Granulocyte-macrophage colony-stimulating factor (GM-CSF), Granulocyte colony-stimulating factor (G-CSF) and Macrophage colony-stimulating factor (M-CSF) were studied. Treatment with KB3495, atorvastatin or the combination resulted in a significant reduction of M-CSF compared to controls (Figure 2A). Similarly the levels of G-CSF were reduced by KB3495 treatment whereas the levels of GM-CSF slightly increased respectively (Table S1 in File S1). Atorvastatin alone or in combination with KB3495 had opposite effects on GM-CSF and G-CSF (Table S1 in File S1). Next we analyzed chemokines that support the recruitment of monocytes into the atherosclerotic plaques. Treatment with KB3495 alone significantly reduced serum levels of monocyte chemotactic protein-1 (MCP-1), involved in the chemokine receptor CCR2 mediated recruitment (Figure 2B), while the chemokines involved in the CCR5 mediated recruitment, such as Rantes or MIP-1α were either slightly increased or not affected, respectively (Table S1 in File S1). Atorvastatin treatment resulted in the induction of MCP-1 and MIP-1α and a reduction in Rantes expression (Figure 2B and Table S1 in File S1). To finally investigate whether macrophage reduction following KB3495 treatment could be associated with an improved immune-inflammatory status, additional markers of the inflammatory status were characterized. Treatment with KB3495 resulted in a significant reduction of IL-1beta, IL-6, TNF alpha and Interferon γ (Figure 2B). The combination of KB3495 with atorvastatin did not result in any additional benefit on the profile of these cytokines (Figure 2B). 

### Hepatic expression of ABCA1 and SR-B1

After 10 weeks of treatment the protein expression of ABCA1 was increased in KB3495 treated animals compared to controls, the atorvastatin and the combination groups had a lower expression compared to controls (Figure S4A). The mRNA expression was 21% (p<0.01; Table 3) lower in the KB3495 group and 35% (p<0.001; Table 3) lower in the combination group. After 25 weeks ABCA1 protein expression was slightly reduced following KB and combination treatment (Figure S4B), mRNA expression was unchanged (Table 3). 

**Table 3:** Hepatic mRNA expression after 10 and 25 weeks of treatment.

	**10 weeks**				**25 weeks**			
	**Ctrl**	**KB**	**Atorva**	**Comb.**	**Ctrl**	**KB**	**Atorva**	**Comb.**
**Abca1**	100±7	79±4**	88±3	65±4***	100	88	69	94
**Abcg5**	100±10	70±6**	123±7	115±5	100±8	74±7*	137±3*	114±4
**Abcg8 Acat2**	100±10 100	96±8 126	156±13** 110	165±11***115	100±9 100	96±7 68	187±12***126	160±15** 141
**Cyp27a1**	100±14	99±5	105±4	112±7	100±12	80±7	90±7	104±9
**Cyp7a1**	100±14	104±13	178±23*	304±36***	100±24	97±11	111±39	121±19
**Cyp8b1**	100±9	115±13	59±7**	64±8**	100±11	107±15	68±10	68±7
**Fgf15#**	100±20	11±4***	177±26***	11±5***	100±44	41±13	114±41	45±12
**Hmgr**	100±14	79±11	82±16	226±35***	100±11	127±11	154±21*	213±31***
**LdlR**	100±8	86±5	83±4*	112±5	100±19	142±9*	97±8	126±8
**Pcsk9**	100±8	86±10	77±10	121±9	100±11	170±55	320±109*	998±299***
**Shp**	100	105	90	80	100	95	61	85
**Srb1**	100±11	90±4	87±4	82±5	100	76	70	72
**Srebp1c**	100±17	54±7*	64±12*	38±6***	100±34	139±19	78±12	109±20
**Srebp2**	100±5	99±5	107±5	134±11**	100±4	125±12	139±10*	175±19***
**Tgh1**	100±9	121±14	23±3***	29±3***	100±35	146±13	72±11	166±33
**Tgh2**	100±9	117±13	10±2***	16±2***	100±31	183±26	29±7**	217±93

Mean values expressed as percent of control ± SEM. In animals treated for 10 weeks n= 10 per group, in animals treated for 25 weeks n=7 in controls, n=9 in the other groups.* = *p* < 0.05; ** = *p* < 0.01; *** = *p* < 0.001. In case of no SEM, only pooled data is analyzed.

# mRNA from intestine.

Protein expression of SR-B1 was not changed in the KB3495 group, neither after 10 nor after 25 weeks. The atorvastatin group showed decreased protein expression following both 10 and 25 weeks. The combination group showed reduced protein expression of SRB1 after 10 weeks, after 25 weeks only minor reductions could be seen (Figures S4A and S4B). The mRNA levels of *Sr-b1* were not changed in either group neither after 10 nor 25 weeks (Table 3). 

### Cholesterol synthesis, transport and excretion

**Figure 3:**
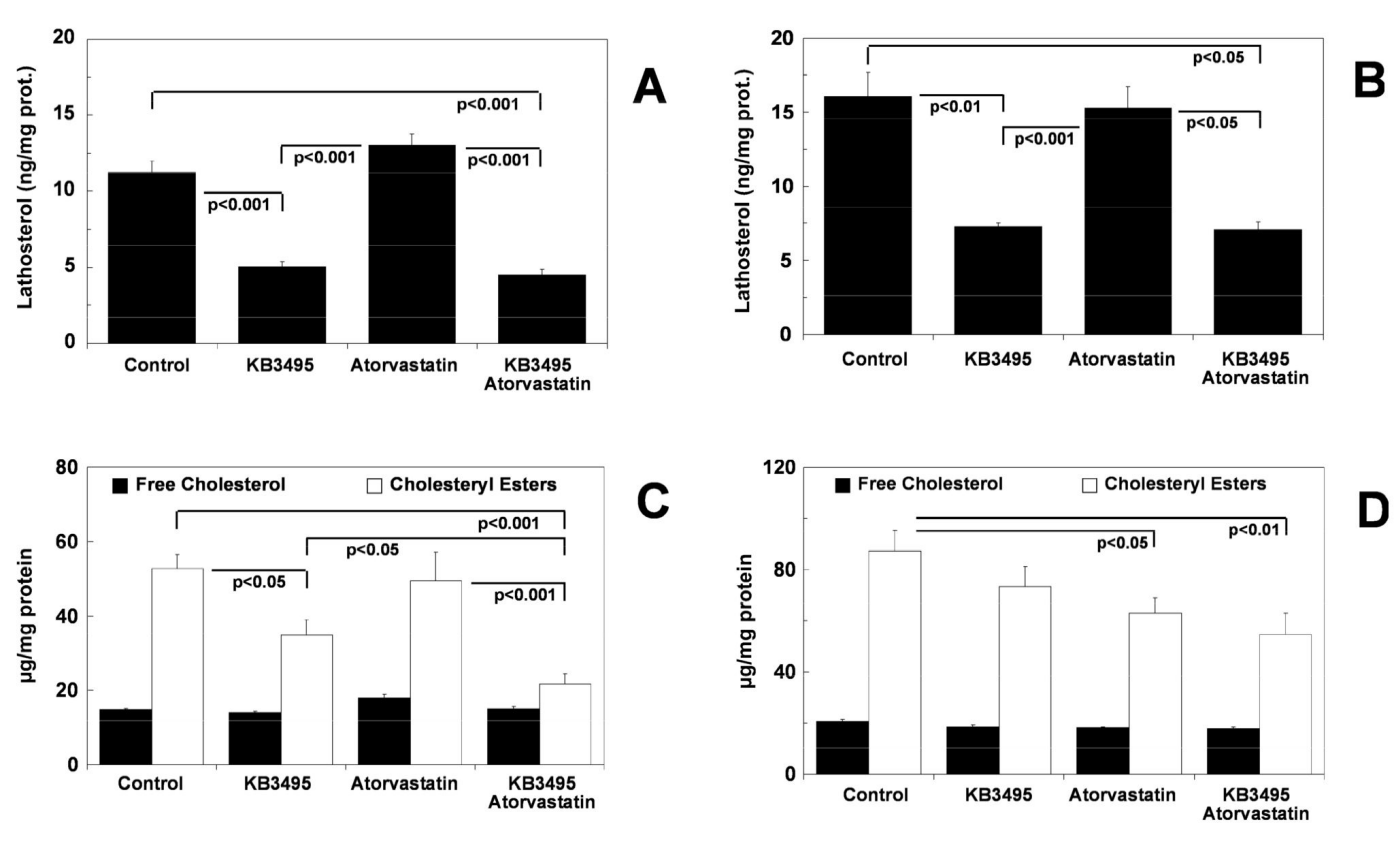
Lipid content in the liver. (A) Lathosterol content in the liver from animals treated for 10 weeks, n=10 per group. (B) Lathosterol content in the liver from animals treated for 25 weeks, n=7 in controls, n=9 in the other groups. (C) Free cholesterol and cholesteryl ester content in liver from animals treated for 10 weeks, n=10 per group. (D) Free cholesterol and cholesteryl ester content in liver from animals treated for 25 weeks, n=7 in controls, n=9 in the other groups. (E) Triglyceride content in liver from animals treated for 10 weeks, n=10 per group. (F) Triglyceride content in liver from animals treated for 25 weeks, n=7 in controls, n=9 in the other groups. All data are corrected for protein content and show mean ± SEM.

Since lower serum cholesterol was not the cause of the reduced atherosclerosis observed in the KB3495-treated animals we investigated if the synthesis, storage and excretion of cholesterol was affected by KB3495. Hepatic lathosterol levels were reduced by approximately 50% in the KB3495 and the combination group following both 10 (p< 0.001; Figure 3A) and 25 (p< 0.01; p< 0.05; Figure 3B) weeks of treatment, indicating a reduced cholesterol synthesis.

After 10 weeks the hepatic CE content was reduced following KB3495 (-34%; p< 0.05) and combination (-59%; p< 0.001) treatment; whereas no differences were observed in FC levels (Figure 3C). Following 25 weeks CE levels in the liver did not show the same pattern as after 10 weeks of treatment. A trend towards reduced levels in the KB3495 group was present, however a statistically significant reduction was only observed in the atorvastatin (p<0.05) and combination groups (p<0.01; Figure 3D).

To investigate the mechanisms behind the changed cholesterol levels in livers, protein and mRNA levels of key genes involved in hepatic cholesterol metabolism were determined. See Table 3 for mRNA results. Following 10 weeks of treatment protein expression for LDLR was decreased in the KB3495 group, this however was not paralleled by similar changes in mRNA expression (Figure S4A; and Table 3). The mRNA expression of *Hmgr* was increased (220%; p< 0.001) in the combination group. Hepatic gene expression of *Pcsk9* did not show any significant changes and had an expression pattern almost identical to that seen for *Ldlr*. Gene expression of *Abcg5* was decreased to 70% (p< 0.01) of controls following KB3495 treatment, no changes was observed in *Abcg8* expression in the KB3495 group. However, *Abcg8* levels were increased to approximately 160% (p< 0.01 resp. 0.001) in the atorvastatin and combination groups. The mRNA levels of triglyceride hydrolase (*Tgh*) *1* and *Tgh2* were not altered by KB3495 but were reduced by approximately 80% and 70% respectively following statin and the combination treatment (p< 0.001). The transcription factor *Srebp1c* was decreased to about 50% in all three groups compared to controls (p< 0.05 for single treatments and p< 0.001 for combination). *Srebp2* levels were increased (134%; p< 0.01) in the combination group but remained stable in the other groups. The mRNA level of *Soat2* was not changed by either treatment nor was the ACAT2 activity (Figure S5; Table 3).

**Figure 4:**
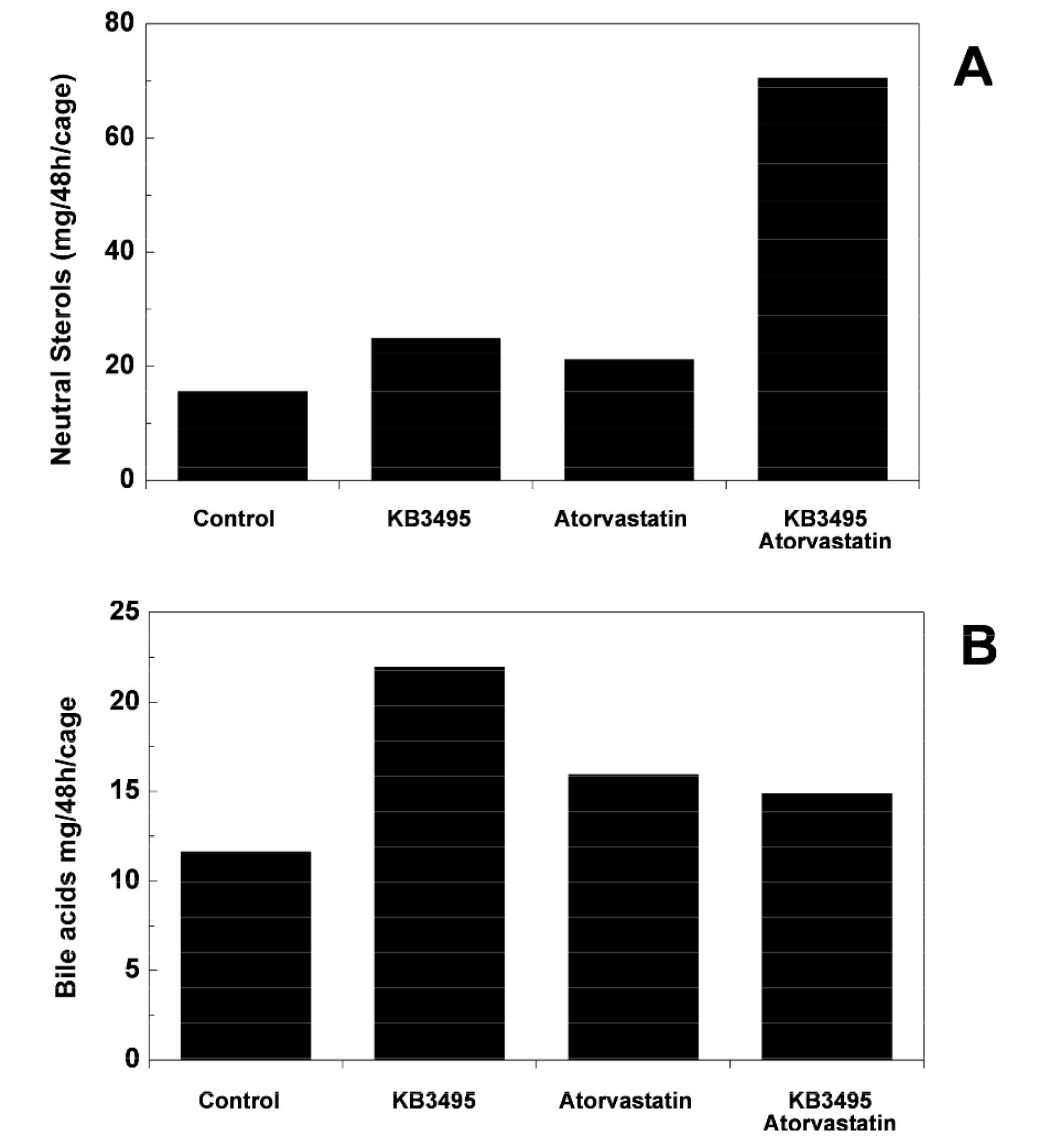
Fecal excretion. (A) Fecal excretion of neutral sterols from animals treated for 10 weeks, feces was collected group wise (pooled) for 48h, n=10 per group. (B) Fecal excretion of bile acids from animals treated for 10 weeks, feces was collected group wise (pooled) for 48h, n=10 per group.

Following 25 weeks of treatment mRNA levels of *Ldlr* was increased to 142% (p<0.05) in the KB3495 group whereas no change was observed in the atorvastatin and combination group. In contrast, *Pcsk9* expression was markedly upregulated following atorvastatin (320%; p<0.05) and combination treatment (998%; p<0.001). No changes were observed in the KB3495 group. Despite the high expression of *Pcsk9* the protein level of LDLR was increased following atorvastatin (171%) and combination treatment (134%; Figure S4B). The mRNA expression of *Hmgr* was increased following atorvastatin (154%; p< 0.05) and combination (213%; p< 0.001) treatment. Gene expression of *Abcg5* and *Abcg8* were not changed from 10 to 25 weeks. mRNA levels of *Tgh1* and *Tgh2* were still reduced by atorvastatin but after 25 weeks there was no longer any reduction in the combination group, 

After 10 weeks of treatment the fecal excretion of neutral sterols was increased in all groups compared to controls, the largest increase was observed in the combination group (350%; Figure 4A).

### BA synthesis, transport and excretion

In order to assess the conversion of cholesterol into BAs we determined the fecal BA excretion. Following 10 weeks of treatment BA excretion was increased by approximately 90% in the KB3495 group compared to controls indicating an increased BA synthesis. Addition of atorvastatin somewhat reduced the effects of KB3495 (Figure 4B). Next, hepatic mRNA expressions for key genes in BA synthesis and transport (10 weeks) were analyzed (Table 3). Expression of *Cyp7a1*, the rate limiting enzyme in the neutral pathway of BA synthesis, increased by 78 % (p< 0.05) following atorvastatin treatment and by 204 % (p< 0.001) after combination treatment. Expression of *Cyp8b1*, *Cyp27a1* and *Shp* was not changed by KB3495 treatment, whereas an almost 50% decrease in *Cyp8b1* (p<0.01) was observed in the atorvastatin group. Recently, FGF15 has been identified as an ileal growth factor involved in the feedback regulation of BA synthesis in the liver [26]. Thus, the expression of *Fgf15* in the ileum was determined. KB3495 alone, or in association with atorvastatin, decreased the mRNA levels to 11% (p<0.001). 

Unfortunately no feces were collected following 25 weeks of treatment which make it difficult to quantify BA synthesis. After 25 weeks the mRNA expression of CYP7A1 was no longer significantly changed, neither was the expression of FGF15 although there was a tendency to reduced FGF15 expression following KB and combination treatment (Table 3). 

### Hepatic triglycerides

Following 10 weeks of treatment hepatic TG levels were not changed by KB3495 but were lowered by statin and combination treatment (p< 0.001 resp. p< 0.01; Figure 3E). After 25 weeks TG levels were increased following KB3495 (p<0.001) and combination treatment (p<0.01; Figure 3F). 

### Skin lipid content after 25 weeks of treatment

**Figure 5:**
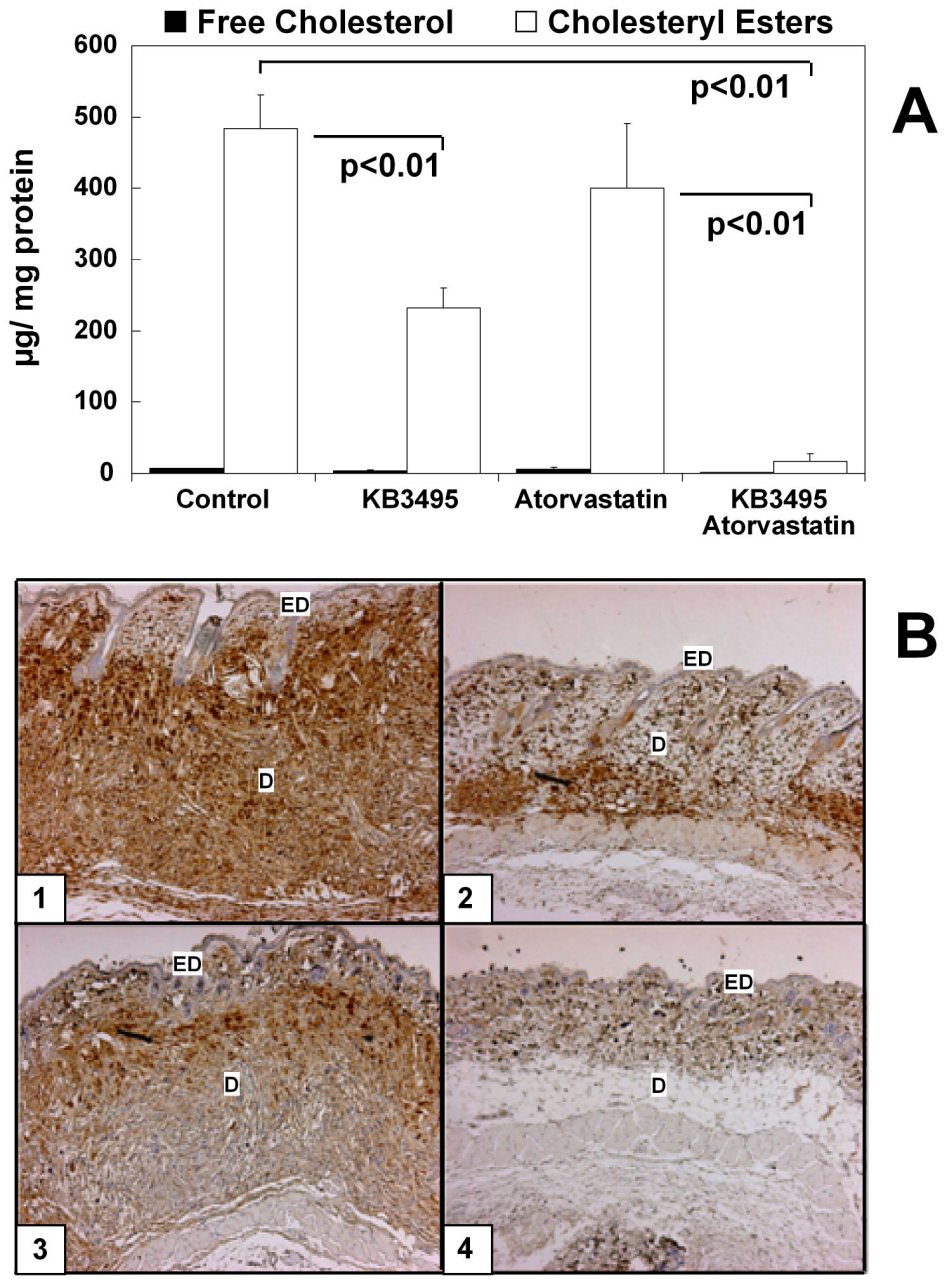
Lipid content in the skin. (A) Free cholesterol and cholesteryl ester content in the skin, data are corrected for protein levels and show mean ± SEM. n=7 in controls, n=9 in KB group, n= 9 in atorvastatin group, n=8 combination group. (B) Histological staining of skin with the specific macrophage Mac-3 marker. ED indicates epidermis, D indicates dermis. 1: control, 2: KB3495, 3: atorvastatin, 4: combination of KB3495 and atorvastatin.

After 25 weeks of a western like diet the skin of the control group appeared thickened and the fur was sparse and in poor condition which was in sharp contrast to the group receiving KB3495 where the fur was glossy and shiny. Therefore skin samples were collected and CE levels were analyzed. In animals receiving KB3495 CE levels were reduced by approximately 50% (p<0.01; Figure 5A), even further reductions were observed in the combination group where the levels were reduced by almost 95% (p<0.01; Figure 5A). Histological analysis revealed that KB3495 had reduced skin thickness (Figure 5B) and a lower percentage of positive staining for macrophages (p<0.001; Table 1).

## Discussion

In this study we investigated the long term effects of KB3495 on the development of atherosclerosis. Using ApoE deficient animals we show that atherosclerosis is reduced following treatment with KB3495. Here we demonstrate for the first time that the anti-atherosclerotic effects are independent of the levels of cholesterol in ApoB-containing lipoproteins. In a previous study where ApoE deficient animals were treated with the thyromimetic compound T-0681 cholesterol levels were reduced in these lipoproteins [27]. We decided to treat animals for 10 and 25 weeks to see whether the effects attributed to thyromimetic compounds would still be present after long term treatment (10 and 25 weeks). 

After 10 weeks of treatment CE levels in aorta were reduced by 50% after KB3495 treatment, following 25 weeks it appears that KB3495 could exert its positive action also in combination with atorvastatin, causing a reduction by 65%. Histochemical analyses of the aortic valves, confirmed the reduction in atherosclerosis. The positive effects of KB3495 after 25 week treatment on atherosclerosis were only evident in the 2-way ANOVA analysis. This was the consequence of a greater variability for CE content observed in the control mice. Treatment with KB3495 was also associated with a reduction of macrophage content in the atherosclerotic plaques and reduced serum levels of IL-1β, TNFalpha, IL-6, Interferon γ, MCP-1 and M-CSF. Interestingly, KB3495 did not only positively affect the cytokines responsible for the maturation and recruitment of monocyte/macrophages in the atherosclerotic plaques but it also attenuated the perpetuation of the inflammatory response. 

Similarly, a reduction of CE in the skin was observed following treatment with KB3495; this reduction was even more prominent in combination with atorvastatin. This dramatic effect on cholesterol deposition in aortas and skin, independent of TC levels in ApoB-containing lipoproteins is remarkable and we are currently addressing the molecular mechanisms behind this effect. Cholesterol accumulation and increased inflammation in the skin of ApoE deficient mice have been reported previously [28,29]. One suggested explanation is an altered migration of dendritic cells in the skin [30]. ApoE deficient mice have an impaired migration of dendritic cells to lymph nodes as compared to wild-type mice and the dendritic cells remaining in epidermis appeared to be activated causing dermal thickening and inflammation. According to studies in LDLr^-/-^ and ApoA-1^-/-^ mice there seems to be a connection between mobilization of skin dentritic cells and cholesterol homeostasis [31]. 

Since KB3495 treatment affects the cholesterol content in both aorta and skin independently of major lipoprotein changes, we investigated whether the substance may have enhanced the hepatic cholesterol metabolism. Following both KB3495 and combination treatment, a reduction of hepatic lathosterol content, indicating a reduced hepatic cholesterol synthesis [32] was observed. This was independent of the duration of the treatments and the effects on hepatic cholesterol synthesis were not paralleled by changes in the hepatic expression of the rate-limiting enzyme *Hmgr*. Similar results was also observed with the TRβ modulator GC-1 [33]. The discrepancy observed between hepatic cholesterol synthesis and *Hmgr* gene expression is nevertheless in line with the known post transcriptional regulation of this gene, a topic reviewed by Ness et al. [34]. 

Earlier studies with selective TR mimetics showed reduced levels of circulating LDL cholesterol and the suggested mechanism is an increased expression of LDLR. T_3_ and TR mimetics have been shown to increase the expression of LDLR in hypophysectomized and intact rats [35,36]. Furthermore, in diet induced obese mice, the *Ldlr* expression has been shown to increase following the administration of T3 and MB07811, however, this effect was transient being visible only at 3 hours and 8 hours after administration and absent after 24 hours [37]. However, the TRβ modulators GC-1 and T-0681 failed to upregulate *Ldlr* in hypercholesterolemic mice [33] and in C57BL/6 or ApoE^-/-^ mice [27], respectively. Thus, the lack of stimulation of *Ldlr* by long-term treatment with KB3495 is not surprising. 

One way to decrease cholesterol accumulation is by stimulation of RCT. This complex process which transfers cholesterol from peripheral cells to the liver for subsequent elimination as BAs and neutral steroids was originally proposed by Glomset more than 40 years ago [38]. Following treatment with KB3495 alone or in combination with atorvastatin, the fecal excretion of neutral sterols increased by approximately 60% and 350%, respectively, demonstrating a net efflux of cholesterol from the body. Previously, the biliary secretion of cholesterol has been shown to be increased in hypothyroid rats upon TH administration [39]. This was later explained by the induction of *Abcg5* and *Abcg8* following T4 substitution [40]. However, we did not observe any increase in the hepatic expression of *Abcg5* and *Abcg8* following KB3495 treatment. Nevertheless, the fecal excretion of neutral sterols was highly induced by KB3495 especially in combination with atorvastatin. Cholesterol can be excreted in the feces independently of the biliary output. ABCG5/G8 knockout mice, which have extremely low concentrations of biliary cholesterol, show a relatively modest reduction in fecal neutral sterols [41]. Furthermore, after LXR induction or hepatic ACAT2 depletion an increase in fecal neutral sterol excretion was observed independently of the biliary cholesterol secretion [42,43]. The above observations can be explained by the newly described trans intestinal cholesterol excretion (TICE) in which enterocytes contribute to fecal neutral sterols by a direct secretion of cholesterol [44]. This study was designed and performed prior the description of TICE and biliary cholesterol secretion rates were unfortunately not measured. 

In order to assess the conversion of cholesterol into BAs, we determined the fecal BA excretion. KB3495 caused a strong increase in BA synthesis, an effect that was in part reduced by addition of atorvastatin, which instead increased the neutral sterol excretion. Interestingly, the effects on BA synthesis were not always paralleled by similar changes in hepatic *Cyp7a1* expression. Previous studies with TH and other TH mimetics (GC-1) have clearly shown that *Cyp7a1* expression is regulated by TH in rodents [33,45,46] and a thyroid receptor responsive element (TRE) has been identified in the promoter region of this gene [47]. The absence of a clear correlation between *Cyp7a1* expression and fecal BA expression in our experiments compared to the study with the GC-1 compound may be due to differences in administration of the drug (orally *vs.* i.p.).

Data from this study indicates that KB3495 causes a reduction in cholesterol synthesis and an increase in BA as well as neutral sterol excretion without affecting serum cholesterol levels in ApoE deficient mice. One explanation for this observation may be that the dyslipidemia of ApoE deficient mice is mainly due to remnant lipoproteins of intestinal origin [25]. Hepatic VLDL secretion reduced in these animals [48]. In ApoE deficient mice, a Western-like diet induces a massive dyslipidemia and the circulating levels of cholesterol and TGs mainly reflect the contribution from the intestine. Thus, it is expected that the inhibition of endogenous cholesterol synthesis *per se* is not sufficient to affect the plasma levels of cholesterol and BAs.

Unlike cholesterol, TG levels were increased following KB3495 treatment. After 10 weeks there was a slight increase in serum TG whereas liver TG was unchanged compared to controls. However, after 25 weeks, serum TG was unchanged but elevated levels were detected in the liver. In atorvastatin treated animals on the other hand, there was no increase in liver TG but serum TG was elevated. Thus, it seems like KB3495 treatment promotes TG storage while atorvastatin treatment increases the transport out to the peripheral tissues. 

## Conclusions

In conclusion, KB3495 reduce atherosclerosis independently of changes in total cholesterol levels in ApoB-containing lipoproteins. Cholesterol levels are reduced by multiple ways; cholesterol synthesis is decreased, formation of BAs, as well as the excretion of fecal BAs and neutral sterols is induced. The beneficial effect on atherosclerosis is also paralleled with decreased inflammation. Whether the reduced inflammation is the consequence of or is the cause for the reduced atherosclerosis is still an open question. 

## Supporting Information

Figure S1 **KB3495.** Structure and IC50 of KB3495.
(TIF)

Figure S2 **Serum lipoproteins.** Serum lipoproteins from animals treated for 10 weeks, the mean curve is shown. (A) Total cholesterol. (B) Free cholesterol. (C) Triglycerides. (D) Phospholipids.
(TIF)

Figure S3 **Serum lipoproteins.** Serum lipoproteins from animals treated for 25 weeks, the mean curve is shown. (A) Total cholesterol. (B) Free cholesterol. (C) Triglycerides. (D) Phospholipids.
(TIF)

Figure S4 **Protein expression.** (A) Protein expression of LDLR, ABCA1 and SRB1 in pooled liver membranes from animals treated for 10 weeks, n=10 per group. (B) Protein expression of LDLR, ABCA1 and SRB1 in pooled liver membranes from animals treated for 25 weeks, n=7 in controls, n=9 in the other groups. Data are expressed as percent of control.
(TIF)

Figure S5 **ACAT activity.** ACAT activity in liver microsomes from animals treated for 10 weeks, n=10 per group. Data are expressed as pmol/min/mg, mean values ± SEM.
(TIF)

File S1 **Supporting Information Methods.** Table S1, Serum concentration of chemokines and cytokines (pg/mL).
(DOCX)

## References

[1] Expert Panel on Detection, Evaluation, and Treatment of High Blood Cholesterol in Adults (2001) Executive Summary of The Third Report of The National Cholesterol Education Program (NCEP) Expert Panel on Detection, Evaluation, And Treatment of High Blood Cholesterol In Adults (Adult Treatment Panel III). JAMA 285: 2486-2497 doi:10.1001/jama.285.19.2486. PubMed: 11368702.

[2] Khera AV, Rader DJ (2010) Future therapeutic directions in reverse cholesterol transport. Curr Atheroscler Rep 12: 73-81. doi:10.1007/s11883-009-0080-0. PubMed: 20425274. 20425274 PMC3315100

[3] Abrams JJ, Grundy SM (1981) Cholesterol metabolism in hypothyroidism and hyperthyroidism in man. J Lipid Res 22: 323-338. PubMed: 7240961. 7240961

[4] Duntas LH (2002) Thyroid disease and lipids. Thyroid 12: 287-293. doi:10.1089/10507250252949405. PubMed: 12034052. 12034052

[5] Bassett JH, Williams GR (2003) The molecular actions of thyroid hormone in bone. Trends Endocrinol Metab 14: 356-364. doi:10.1016/S1043-2760(03)00144-9. PubMed: 14516933. 14516933

[6] Klein I, Ojamaa K (2001) Thyroid hormone and the cardiovascular system. N Engl J Med 344: 501-509. doi:10.1056/NEJM200102153440707. PubMed: 11172193. 11172193

[7] Bookout AL, Jeong Y, Downes M, Yu RT, Evans RM et al. (2006) Anatomical profiling of nuclear receptor expression reveals a hierarchical transcriptional network. Cell 126: 789-799. doi:10.1016/j.cell.2006.06.049. PubMed: 16923397. 16923397 PMC6211849

[8] Weiss RE, Korcarz C, Chassande O, Cua K, Sadow PM et al. (2002) Thyroid hormone and cardiac function in mice deficient in thyroid hormone receptor-alpha or -beta: an echocardiograph study. Am J Physiol Endocrinol Metab 283: E428-E435. PubMed: 12169435. 12169435 10.1152/ajpendo.00019.2002

[9] Berkenstam A, Kristensen J, Mellström K, Carlsson B, Malm J et al. (2008) The thyroid hormone mimetic compound KB2115 lowers plasma LDL cholesterol and stimulates bile acid synthesis without cardiac effects in humans. Proc Natl Acad Sci U S A 105: 663-667. doi:10.1073/pnas.0705286104. PubMed: 18160532. 18160532 PMC2206593

[10] Ladenson PW, Kristensen JD, Ridgway EC, Olsson AG, Carlsson B et al. (2010) Use of the thyroid hormone analogue eprotirome in statin-treated dyslipidemia. N Engl J Med 362: 906-916. doi:10.1056/NEJMoa0905633. PubMed: 20220185. 20220185

[11] Baxter JD, Webb P (2009) Thyroid hormone mimetics: potential applications in atherosclerosis, obesity and type 2 diabetes. Nat Rev Drug Discov 8: 308-320. doi:10.1038/nrd2830. PubMed: 19337272. 19337272

[12] Tancevski I, Eller P, Patsch JR, Ritsch A (2009) The resurgence of thyromimetics as lipid-modifying agents. Curr Opin Investig Drugs 10: 912-918. PubMed: 19705333. PMC2993058 19705333

[13] Grover GJ, Kelly J, Malm J (2007) Thyroid hormone receptor subtype-β-selective agonists as potential treatments for metabolic syndrome. Future Lipidology. Future Medicine 2: 641-649.

[14] Chiellini G, Apriletti JW, Yoshihara HA, Baxter JD, Ribeiro RC et al. (1998) A high-affinity subtype-selective agonist ligand for the thyroid hormone receptor. Chem Biol 5: 299-306. doi:10.1016/S1074-5521(98)90168-5. PubMed: 9653548. 9653548

[15] Grover GJ, Mellström K, Ye L, Malm J, Li YL et al. (2003) Selective thyroid hormone receptor-beta activation: a strategy for reduction of weight, cholesterol, and lipoprotein (a) with reduced cardiovascular liability. Proc Natl Acad Sci U S A 100: 10067-10072. doi:10.1073/pnas.1633737100. PubMed: 12888625. 12888625 PMC187768

[16] Parini P, Johansson L, Bröijersén A, Angelin B, Rudling M (2006) Lipoprotein profiles in plasma and interstitial fluid analyzed with an automated gel-filtration system. Eur J Clin Invest 36: 98-104. doi:10.1111/j.1365-2362.2006.01597.x. PubMed: 16436091. 16436091

[17] Folch J, Lees M, Sloane Stanley GH (1957) A simple method for the isolation and purification of total lipides from animal tissues. J Biol Chem 226: 497-509. PubMed: 13428781. 13428781

[18] Björkhem I, Blomstrand R, Svensson L (1974) Serum cholesterol determination by mass fragmentography. Clin Chim Acta 54: 185-193. doi:10.1016/0009-8981(74)90236-8. PubMed: 4854256. 4854256

[19] Lund E, Sisfontes L, Reihner E, Bjorkhem I (1989) Determination of serum levels of unesterified lathosterol by isotope dilution-mass spectrometry. Scand J Clin Lab Invest 49: 165-171. doi:10.3109/00365518909105417. PubMed: 2520369. 2520369

[20] Parini P, Davis M, Lada AT, Erickson SK, Wright TL et al. (2004) ACAT2 is localized to hepatocytes and is the major cholesterol-esterifying enzyme in human liver. Circulation 110: 2017-2023. doi:10.1161/01.CIR.0000143163.76212.0B. PubMed: 15451793. 15451793

[21] Miettinen TA (1982) Gas-liquid chromatographic determination of fecal neutral sterols using a capillary column. Clin Chim Acta 124: 245-248. doi:10.1016/0009-8981(82)90393-X. PubMed: 7139944. 7139944

[22] Grundy SM, Ahrens EH Jr., Miettinen TA (1965) Quantitative Isolation and Gas--Liquid Chromatographic Analysis of Total Fecal Bile Acids. J Lipid Res 6: 397-410. PubMed: 14336211. 14336211

[23] George W. Snedecor WGC (1980) Statistical Methods. Ames: Iowa State University Press (Ames, Iowa).

[24] Rudel LL, Kelley K, Sawyer JK, Shah R, Wilson MD (1998) Dietary monounsaturated fatty acids promote aortic atherosclerosis in LDL receptor-null, human ApoB100-overexpressing transgenic mice. Arterioscler Thromb Vasc Biol 18: 1818-1827. doi:10.1161/01.ATV.18.11.1818. PubMed: 9812923. 9812923

[25] Plump AS, Smith JD, Hayek T, Aalto-Setälä K, Walsh A et al. (1992) Severe hypercholesterolemia and atherosclerosis in apolipoprotein E-deficient mice created by homologous recombination in ES cells. Cell 71: 343-353. doi:10.1016/0092-8674(92)90362-G. PubMed: 1423598. 1423598

[26] Inagaki T, Choi M, Moschetta A, Peng L, Cummins CL et al. (2005) Fibroblast growth factor 15 functions as an enterohepatic signal to regulate bile acid homeostasis. Cell Metab 2: 217-225. doi:10.1016/j.cmet.2005.09.001. PubMed: 16213224. 16213224

[27] Tancevski I, Demetz E, Eller P, Duwensee K, Hoefer J et al. (2010) The liver-selective thyromimetic T-0681 influences reverse cholesterol transport and atherosclerosis development in mice. PLOS ONE 5: e8722. doi:10.1371/journal.pone.0008722. PubMed: 20090943. 20090943 PMC2806908

[28] Feingold KR, Elias PM, Mao-Qiang M, Fartasch M, Zhang SH et al. (1995) Apolipoprotein E deficiency leads to cutaneous foam cell formation in mice. J Invest Dermatol 104: 246-250. doi:10.1111/1523-1747.ep12612790. PubMed: 7829881. 7829881

[29] van Ree JH, Gijbels MJ, van den Broek WJ, Hofker MH, Havekes LM (1995) Atypical xanthomatosis in apolipoprotein E-deficient mice after cholesterol feeding. Atherosclerosis 112: 237-243. doi:10.1016/0021-9150(94)05419-J. PubMed: 7772082. 7772082

[30] Angeli V, Llodrá J, Rong JX, Satoh K, Ishii S et al. (2004) Dyslipidemia associated with atherosclerotic disease systemically alters dendritic cell mobilization. Immunity 21: 561-574. doi:10.1016/j.immuni.2004.09.003. PubMed: 15485633. 15485633

[31] Zabalawi M, Bharadwaj M, Horton H, Cline M, Willingham M et al. (2007) Inflammation and skin cholesterol in LDLr-/-, apoA-I-/- mice: link between cholesterol homeostasis and self-tolerance? J Lipid Res 48: 52-65. PubMed: 17071966. 17071966 10.1194/jlr.M600370-JLR200

[32] Murphy C, Parini P, Wang J, Björkhem I, Eggertsen G et al. (2005) Cholic acid as key regulator of cholesterol synthesis, intestinal absorption and hepatic storage in mice. Biochim Biophys Acta 1735: 167-175. doi:10.1016/j.bbalip.2005.06.001. PubMed: 15994119. 15994119

[33] Johansson L, Rudling M, Scanlan TS, Lundåsen T, Webb P et al. (2005) Selective thyroid receptor modulation by GC-1 reduces serum lipids and stimulates steps of reverse cholesterol transport in euthyroid mice. Proc Natl Acad Sci U S A 102: 10297-10302. doi:10.1073/pnas.0504379102. PubMed: 16006512. 16006512 PMC1177400

[34] Ness GC, Chambers CM (2000) Feedback and hormonal regulation of hepatic 3-hydroxy-3-methylglutaryl coenzyme A reductase: the concept of cholesterol buffering capacity. Proc Soc Exp Biol Med 224: 8-19. doi:10.1046/j.1525-1373.2000.22359.x. PubMed: 10782041. 10782041

[35] Staels B, Van Tol A, Chan L, Will H, Verhoeven G et al. (1990) Alterations in thyroid status modulate apolipoprotein, hepatic triglyceride lipase, and low density lipoprotein receptor in rats. Endocrinology 127: 1144-1152. doi:10.1210/endo-127-3-1144. PubMed: 2387252. 2387252

[36] Lopez D, Abisambra Socarrás JF, Bedi M, Ness GC (2007) Activation of the hepatic LDL receptor promoter by thyroid hormone. Biochim Biophys Acta 1771: 1216-1225. doi:10.1016/j.bbalip.2007.05.001. PubMed: 17572141. 17572141

[37] Erion MD, Cable EE, Ito BR, Jiang H, Fujitaki JM et al. (2007) Targeting thyroid hormone receptor-beta agonists to the liver reduces cholesterol and triglycerides and improves the therapeutic index. Proc Natl Acad Sci U S A 104: 15490-15495. doi:10.1073/pnas.0702759104. PubMed: 17878314. 17878314 PMC1978486

[38] Glomset JA (1968) The plasma lecithins:cholesterol acyltransferase reaction. J Lipid Res 9: 155-167. PubMed: 4868699. 4868699

[39] Gebhard RL, Stone BG, Andreini JP, Duane WC, Evans CD et al. (1992) Thyroid hormone differentially augments biliary sterol secretion in the rat. I. The isolated-perfused liver model. J Lipid Res 33: 1459-1466. PubMed: 1431570. 1431570

[40] Gälman C, Bonde Y, Matasconi M, Angelin B, Rudling M (2008) Dramatically increased intestinal absorption of cholesterol following hypophysectomy is normalized by thyroid hormone. Gastroenterology 134: 1127-1136. doi:10.1053/j.gastro.2008.01.032. PubMed: 18395092. 18395092

[41] Yu L, Li-Hawkins J, Hammer RE, Berge KE, Horton JD et al. (2002) Overexpression of ABCG5 and ABCG8 promotes biliary cholesterol secretion and reduces fractional absorption of dietary cholesterol. J Clin Invest 110: 671-680. doi:10.1172/JCI200216001. PubMed: 12208868. 12208868 PMC151111

[42] Kruit JK, Plösch T, Havinga R, Boverhof R, Groot PH et al. (2005) Increased fecal neutral sterol loss upon liver X receptor activation is independent of biliary sterol secretion in mice. Gastroenterology 128: 147-156. doi:10.1053/j.gastro.2004.10.006. PubMed: 15633131. 15633131

[43] Brown JM, Bell TA 3rd, Alger HM, Sawyer JK, Smith TL et al. (2008) Targeted depletion of hepatic ACAT2-driven cholesterol esterification reveals a non-biliary route for fecal neutral sterol loss. J Biol Chem 283: 10522-10534. doi:10.1074/jbc.M707659200. PubMed: 18281279. 18281279 PMC2447638

[44] van der Velde AE, Vrins CL, van den Oever K, Kunne C, Oude Elferink RP et al. (2007) Direct intestinal cholesterol secretion contributes significantly to total fecal neutral sterol excretion in mice. Gastroenterology 133: 967-975. doi:10.1053/j.gastro.2007.06.019. PubMed: 17854600. 17854600

[45] Ness GC, Pendleton LC, Li YC, Chiang JY (1990) Effect of thyroid hormone on hepatic cholesterol 7 alpha hydroxylase, LDL receptor, HMG-CoA reductase, farnesyl pyrophosphate synthetase and apolipoprotein A-I mRNA levels in hypophysectomized rats. Biochem Biophys Res Commun 172: 1150-1156. doi:10.1016/0006-291X(90)91568-D. PubMed: 2123100. 2123100

[46] Pandak WM, Heuman DM, Redford K, Stravitz RT, Chiang JY et al. (1997) Hormonal regulation of cholesterol 7alpha-hydroxylase specific activity, mRNA levels, and transcriptional activity in vivo in the rat. J Lipid Res 38: 2483-2491. PubMed: 9458272. 9458272

[47] Shin DJ, Plateroti M, Samarut J, Osborne TF (2006) Two uniquely arranged thyroid hormone response elements in the far upstream 5' flanking region confer direct thyroid hormone regulation to the murine cholesterol 7alpha hydroxylase gene. Nucleic Acids Res 34: 3853-3861. doi:10.1093/nar/gkl506. PubMed: 16899449. 16899449 PMC1557806

[48] Kuipers F, Jong MC, Lin Y, Eck M, Havinga R et al. (1997) Impaired secretion of very low density lipoprotein-triglycerides by apolipoprotein E- deficient mouse hepatocytes. J Clin Invest 100: 2915-2922. doi:10.1172/JCI119841. PubMed: 9389759. 9389759 PMC508499

