# Supplementary figures and images for: The Thyroid Receptor Modulator KB3495 Reduces Atherosclerosis Independently of Total Cholesterol in the Circulation in ApoE Deficient Mice
Source: PLoS One. 2013 Dec 4;8(12):e78534. doi: 10.1371/journal.pone.0078534 (PMC3850901; doi:10.1371/journal.pone.0078534)

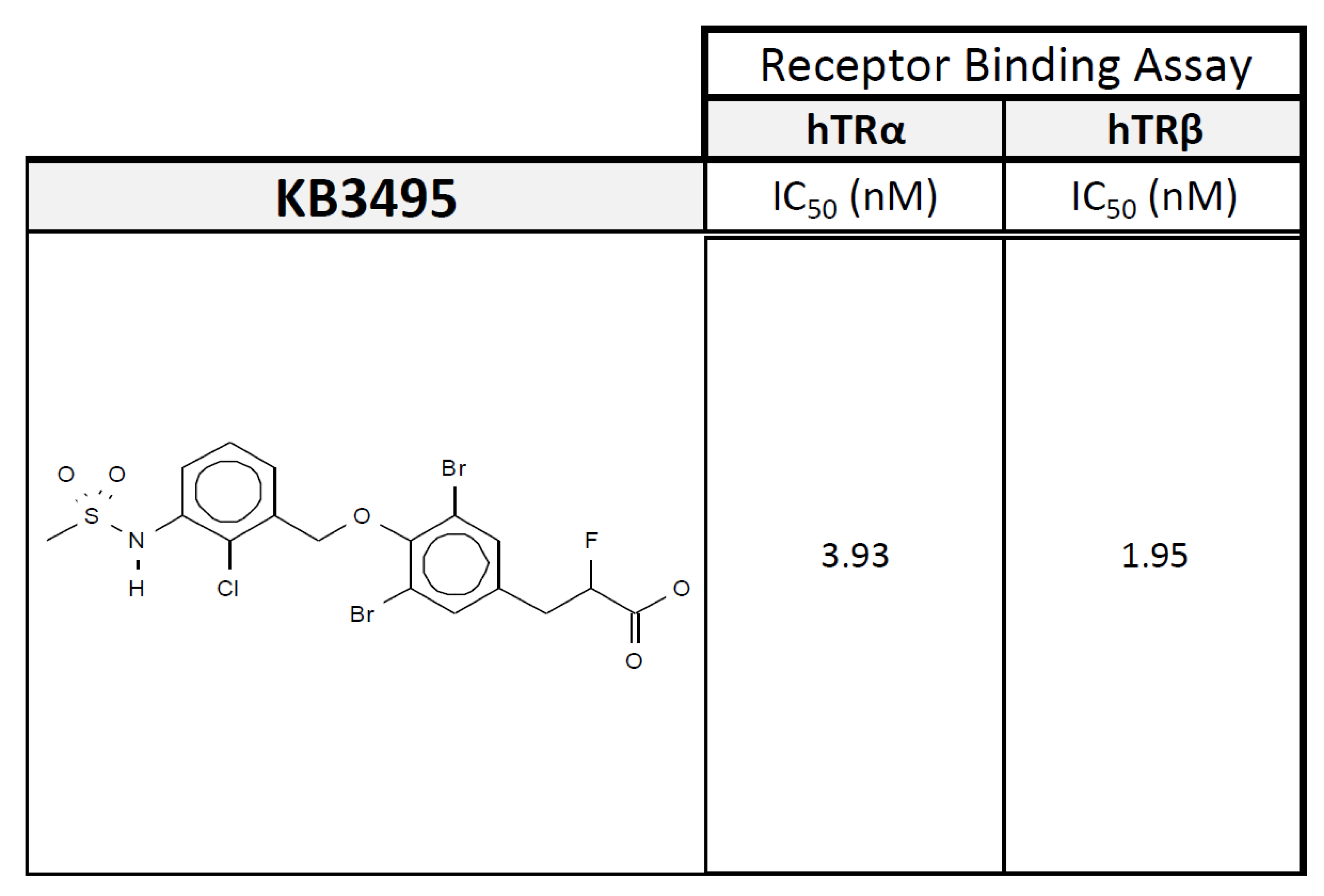

Supplement: Figure S1 — KB3495. Structure and IC50 of KB3495. (TIF) [file pone.0078534.s001.tif]

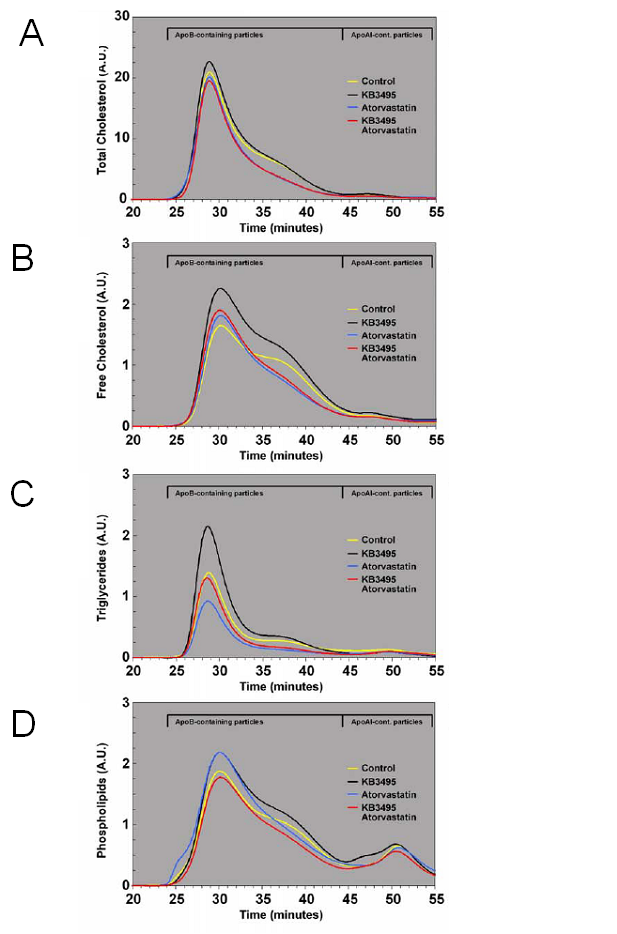

Supplement: Figure S2 — Serum lipoproteins. Serum lipoproteins from animals treated for 10 weeks, the mean curve is shown. (A) Total cholesterol. (B) Free cholesterol. (C) Triglycerides. (D) Phospholipids. (TIF) [file pone.0078534.s002.tif]

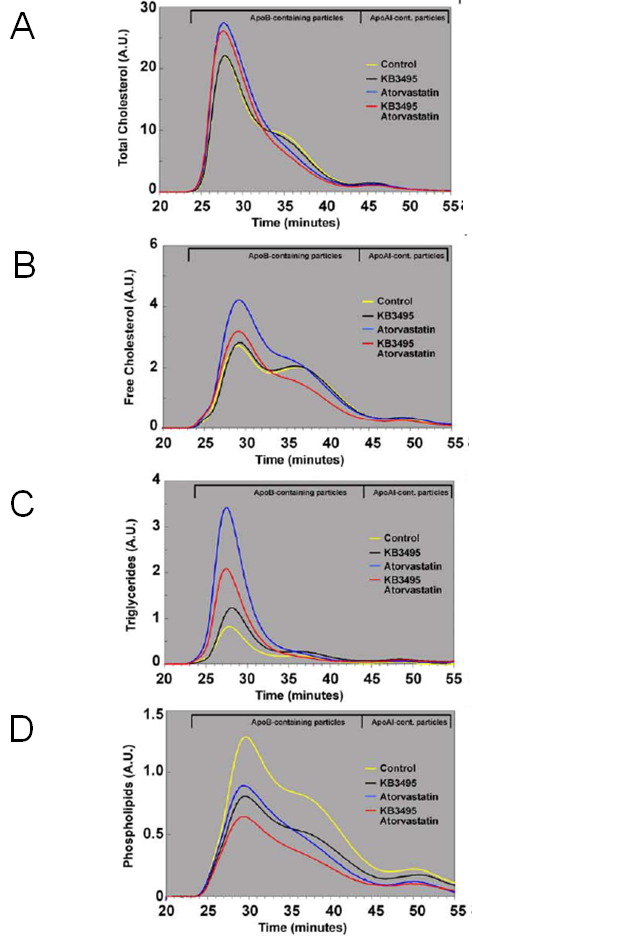

Supplement: Figure S3 — Serum lipoproteins. Serum lipoproteins from animals treated for 25 weeks, the mean curve is shown. (A) Total cholesterol. (B) Free cholesterol. (C) Triglycerides. (D) Phospholipids. (TIF) [file pone.0078534.s003.tif]

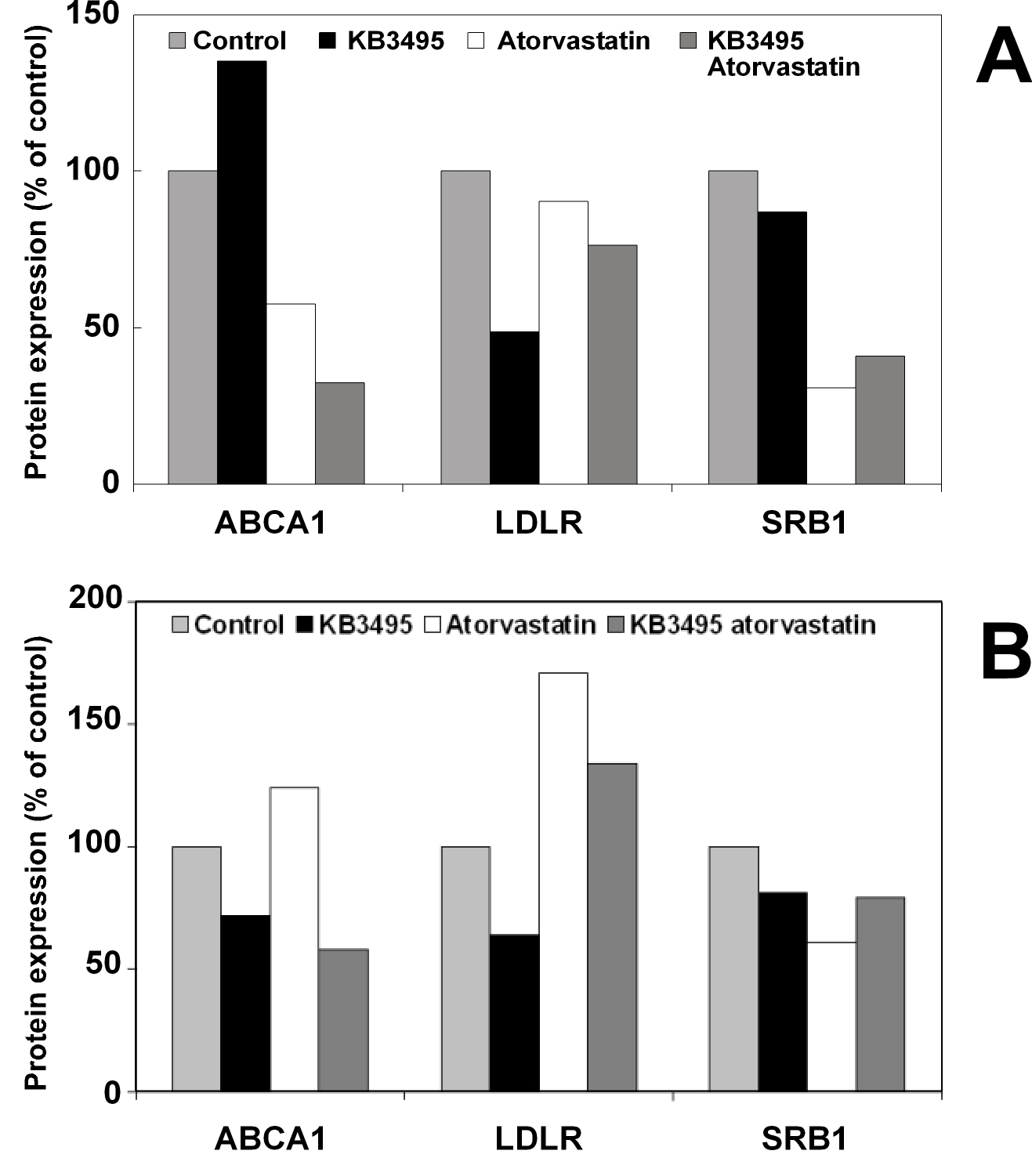

Supplement: Figure S4 — Protein expression. (A) Protein expression of LDLR, ABCA1 and SRB1 in pooled liver membranes from animals treated for 10 weeks, n=10 per group. (B) Protein expression of LDLR, ABCA1 and SRB1 in pooled liver membranes from animals treated for 25 weeks, n=7 in controls, n=9 in the other groups. Data are expressed as percent of control. (TIF) [file pone.0078534.s004.tif]
