# Supplementary material for: The Thyroid Receptor Modulator KB3495 Reduces Atherosclerosis Independently of Total Cholesterol in the Circulation in ApoE Deficient Mice
Source: PLoS One. 2013 Dec 4;8(12):e78534. doi: 10.1371/journal.pone.0078534 (PMC3850901; doi:10.1371/journal.pone.0078534)
Supplement: File S1 — Supporting Information Methods. Table S1, Serum concentration of chemokines and cytokines (pg/mL). (DOCX) [file pone.0078534.s006.docx]

**Supporting Information**

***Methods***

***Cholesterol Analysis***

Total and free cholesterol were determined by isotope dilution-mass spectrometry. Cholesterol was purified using an Isolute-MF C18 cartridge (International Sorbent Technology) prior to derivatization with a trimethylsilane reagent. For the analysis of total cholesterol, cholesterol esters were hydrolyzed in presence of KOH and ethanol prior to purification. D6-labelled cholesterol was used as internal standard.

***Isolation of Hepatic Membranes and Analysis of Protein Expression***

Liver tissue were homogenized in 20 mmol/L Tris-HCl, pH 7.5 / 2 mmol/L CaCl_2_ / 0.25 mol/L sucrose. Homogenates were centrifuged at 4^o^C for 10 min at 2000g. Supernatants were centrifuged at 110 000g for 45 min at 4^o^C. Pellets were suspended in 80 mmol/L NaCl / 50 mmol/L Tris-HCl, pH 8 / 2 mmol/L CaCl_2_ / 1% TritonX-100 and then centrifuged at 4^o^C for 10 min at 20 000g. The final supernatant was collected. After protein determination membranes were pooled groupwise. Pooled membranes (20, 30, 40 µg) were separated on 3-8% Tris-acetate gels (NuPAGE, Invitrogen). Proteins were transferred to nitrocellulose filter. ABCA1 protein was detected with mouse mAb (1:1000; Abcam) and as secondary antibody a peroxidase-conjugated goat anti-mouse immunoglobulin (1:20 000; Pierce). LDLR protein was detected with rabbit pAb (1:1000; Cayman) and a peroxidase-conjugated sheep anti-rabbit antibody (1:10 000; Immunokemi). SR-B1 was detected with rabbit antibody mAb (1:6000; Abcam) and a peroxidase-conjugated donkey anti-rabbit antibody (1:50 000; GE Healthcare). The specific bands were detected using a BioRad Universal Hood II (BioRad) and quantified by BioRad Quantity One software (BioRad). Signals were plotted by mg loaded protein and the slopes were calculated by method of least square. The slope of the control group was set to 100%.

***Table S1***

Serum concentration of chemokines and cytokines (pg/mL).

|  | **G-CSF** | **GM-CSF** | **Rantes** | **MIP-1α** |
| --- | --- | --- | --- | --- |
| **Ctrl** | 725 ± 1.3 | 43 ± 0.9 | 25 ± 0.4 | 25 ± 0.6 |
| **KB** | 699 ± 2.5** | 52 ± 0.9** | 29 ± 0.6** | 24 ± 0.4 |
| **Atorva** | 1198 ± 2.0*** | 42 ± 0.9** | 9.6 ± 0.3*** | 40 ± 1.1*** |
| **Comb.** | 448 ± 1.6 ** | 89 ± 0.4*** | 24 ± 0.9 | 59 ± 1.0*** |

Ctrl: controls; KB: treatment with KB3495; Atorva: treatment with atorvastatin; Comb.: treatment with KB3495 and atorvastatin in combination. Mean values (5 animals /group) ± SEM.
Contrast *vs.* Ctrl ** = *p* < 0.01; *** = *p* < 0.001.
